# Transcriptome-wide analysis of pseudouridylation in *Drosophila melanogaster*

**DOI:** 10.1093/g3journal/jkac333

**Published:** 2022-12-19

**Authors:** Wan Song, Ram Podicheti, Douglas B Rusch, William Daniel Tracey

**Affiliations:** Gill Center for Biomolecular Research, Indiana University, Bloomington, IN 47405, USA; Department of Biology, Indiana University, Bloomington, IN 47405, USA; Center for Genomics and Bioinformatics, Indiana University, Bloomington, IN 47405, USA; Center for Genomics and Bioinformatics, Indiana University, Bloomington, IN 47405, USA; Gill Center for Biomolecular Research, Indiana University, Bloomington, IN 47405, USA; Department of Biology, Indiana University, Bloomington, IN 47405, USA

**Keywords:** *Drosophila melanogaster*, pseudouridine, transcriptome, Psi-seq, CMC, RluA-2 target, differential expression

## Abstract

Pseudouridine (Psi) is one of the most frequent post-transcriptional modification of RNA. Enzymatic Psi modification occurs on rRNA, snRNA, snoRNA, tRNA, and non-coding RNA and has recently been discovered on mRNA. Transcriptome-wide detection of Psi (Psi-seq) has yet to be performed for the widely studied model organism *Drosophila melanogaster*. Here, we optimized Psi-seq analysis for this species and have identified thousands of Psi modifications throughout the female fly head transcriptome. We find that Psi is widespread on both cellular and mitochondrial rRNAs. In addition, more than a thousand Psi sites were found on mRNAs. When pseudouridylated, mRNAs frequently had many Psi sites. Many mRNA Psi sites are present in genes encoding for ribosomal proteins, and many are found in mitochondrial encoded RNAs, further implicating the importance of pseudouridylation for ribosome and mitochondrial function. The 7SLRNA of the signal recognition particle is the non-coding RNA most enriched for Psi. The 3 mRNAs most enriched for Psi encode highly expressed yolk proteins (Yp1, Yp2, and Yp3). By comparing the pseudouridine profiles in the *RluA-2* mutant and the *w^1118^* control genotype, we identified Psi sites that were missing in the mutant RNA as potential RluA-2 targets. Finally, differential gene expression analysis of the mutant transcriptome indicates a major impact of loss of RluA-2 on the ribosome and translational machinery.

## Introduction

Pseudouridylation is one of the earliest discovered and most abundant post-transcriptional RNA modifications. Pseudouridine (Psi), the C5-glycoside isomer of uridine occurs at specific nucleotide residues in rRNAs, tRNAs, snRNAs, snoRNAs, and other ncRNAs (Ge and Yu 2013) and more recently Psi has also been found in mRNA (Carlile *et al*. 2014; Lovejoy *et al*. 2014; Schwartz *et al*. 2014; Li *et al*. 2015). Psi sites within RNAs are thought to have various functions depending on the molecule. Psi sites in tRNAs are present in the anticodon stem-loop, D-stem, and other conserved sites and contribute to the stabilization of tertiary structure. In rRNA, the known Psi sites are concentrated near the decoding site, the peptidyl transferase center, and at sites of interaction with ribosomal proteins, where they are thought to be important for ribosome assembly and protein synthesis (Charette and Gray 2000). In the non-coding RNA (ncRNA) of telomerase, a functionally important Psi residue is located in a region involved in telomerase reverse transcriptase (RT) binding (Penzo *et al*. 2017). Incorporation of Psi into therapeutic mRNAs results in a reduced immune response to the RNA and enhanced protein expression (Kauffman *et al*. 2016).

Experimental detection of pseudouridine residues has been hindered and complicated by the fact that Psi and uridine share the same molecular mass and similar base-pairing properties upon reverse transcription. The most frequently used method to detect this nucleoside is to treat RNA with cyclohexyl-*N*′-(2-morpholinoethyl)-carboiimide metho-*p*-toluene sulfonate (CMCT). CMCT reacts with uridine- and guanosine-like nucleotides and subsequent alkaline treatment of the reacted RNA hydrolyzes CMC adducts to G and U but does not hydrolyze CMC-Psi mono-adducts. The bulky CMC moiety blocks primer extension (Ho and Gilham 1967) and this block may be directly detected with polyacrylamide gel electrophoresis and autoradiography in highly abundant RNA species such as rRNA. Pioneering work by the Ofengand group used this reaction to detect highly conserved Psi sites in rRNA in species ranging from prokaryotes to eukaryotes (Bakin and Ofengand 1993).

More recently, next generation RNA sequencing approaches have been adopted for detection of pseudouridines in lower abundance transcripts. The majority of these methods also depend on CMCT treatment since the CMC adduct conjugated to Psi prevents readthrough by RT. Bioinformatic analyses of CMC-treated libraries identify sites of pseudouridylation where reverse transcription is more frequently terminated relative to reads in untreated libraries. This Psi-seq approach (also known as “pseudo-seq,” “Ψ-seq,” or “CeU-Seq”) has been employed for global transcriptome-wide identification of pseudouridylation sites in yeast (Carlile *et al*. 2014; Lovejoy *et al*. 2014; Schwartz *et al*. 2014), human cell lines (Carlile *et al*. 2014; Schwartz *et al*. 2014; Li *et al*. 2015), mouse (Li *et al*. 2015), *Toxoplasma gondii* (Nakamoto *et al*. 2017), and *Arabidopsis thaliana* (Sun *et al*. 2019).

The post-transcriptional isomerization of uridine to pseudouridine is catalyzed by 6 families of pseudouridine synthases (TruA, TruB, TruD, RsuA, RluA, and Pus10). The archaeal and eukaryotic TruB homologues function with auxiliary guide RNAs, while the rest act as stand-alone enzymes (Ofengand 2002; Hamma and Ferre-D’Amare 2006). Some pseudouridine synthases have highly conserved and specific substrates, whereas others show more broad and complex targets (Rintala-Dempsey and Kothe 2017). In the *Drosophila melanogaster* genome, there are 9 proteins with an annotated pseudouridine synthase domain. *Drosophila* nucleolar protein 60B (Nop60B), also known as *minifly* (*mfl*), is a well-studied homolog of human box H/ACA RNP dyskerin (mouse NAP57 and yeast Cbf5). Nop60B/Mfl is essential for *Drosophila* viability and fertility as it is required for ribosome biogenesis and the *mfl* mutant has reduced pseudouridylation at several sites of 28S and 18S rRNA (Phillips *et al*. 1998; Giordano *et al*. 1999; Breznak *et al.* 2022). Two other pseudouridine synthases (RluA-1 and RluA-2) have been studied for their roles in the negative regulation of nociception (Song *et al*. 2020). RluA-1 is primarily expressed in multidendritic sensory neurons including the larval nociceptors whereas RluA-2 is expressed ubiquitously. RluA-1 and RluA-2 belong to RluA family which have complex substrate specificity in yeast and bacteria (Hoang *et al*. 2006).

Here, we have applied the Psi-seq technique to identify sites of pseudouridylation in *D. melanogaster* female head transcriptome. In addition, we have investigated the pseudouridine and transcriptional profile in animals lacking the ubiquitously expressed RluA family member RluA-2. Our results suggest a major role for RluA-2 for pseudouridylation across a variety of RNA classes as well as large changes in gene expression that occur in animals lacking RluA-2 function.

## Materials and methods

### Fly strains and raising conditions

The isogenic *w^1118^* strain used as the control genotype was obtained from Bloomington stock center. The mutant of *RluA-2^del-FRT^* used in the study was a complete deletion of the locus that was generated and previously described (Song *et al*. 2020). All flies were reared on yeast, corn meal food in bottles, or vials at an incubator with controlled temperature (25°C) and humidity (70%) on a 12 h light/12 h dark cycle.

### RNA extraction from adult head tissue

For Experiment 1 (Exp 1), newly eclosed virgins from isogenized *w^1118^* (iso*w^1118^*) were collected twice every day (once in the morning and once in the afternoon) with <8 h in between. For Experiment 2 (Exp 2), newly eclosed virgins from iso*w^1118^* and homozygous *RluA-2^del-FRT^* (Song *et al*. 2020) were collected in the similar way. The flies were put into vials with yeast and Bloomington recipe fly medium (20–30 flies in each vial) and aged for 1 day in the incubator. The flies were then snap frozen in liquid nitrogen and kept at −80°C for 3 days until sufficient number of flies from each genotype had been collected. The frozen flies were then decapitated and 100 heads per replicate homogenized in TRIzol reagent (Invitrogen). Total RNA was prepared according to the manufacturers' recommendations.

### In vitro transcription of RNA “spike-in”

The RNA spike-in sequence containing a single pseudouridine at position 43 as used previously (Schwartz *et al*. 2014) was in vitro transcribed from dsDNA template (synthesized as gBlocks gene fragment from IDT). In vitro transcription was performed in a volume of 20 μl with MEGAshortscript T7 transcription kit (AM1354; Invitrogen), using 75 nmol each of GTP, CTP, ATP, and Psi-TP (TriLink Biotechnologies). The RNA product was purified with MEGAclear Transcription Clean-Up kit (AM1908; Invitrogen) before adding into the Psi-seq libraries.

### CMCT treatment and RNA-seq library preparation

Polyadenylated RNA was enriched from total RNA using 2 rounds of Oligo(dT) dynabeads (Invitrogen) according to manufacturer's protocol. To each polyA-RNA sample, spike-in RNA (∼3 μg) was added and then the samples were split into 2 equal samples for CMCT or mock treatment. CMCT treatment was carried out essentially as described (Bakin and Ofengand 1993, 1995). However, when we used the exact conditions of prior studies (Schwartz *et al*. 2014), we failed to obtain a distinctive signal of termination fold change at the expected Psi position of our spiked in RNA (data not shown). Therefore, to optimize the CMC treatment and pseudouridylation detection efficiency for our Psi-seq, we tested the impact of several main factors affecting the efficiency of CMCT derivatization, including fragmentation before CMC treatment as used previously (Carlile *et al*. 2014; Li *et al*. 2015), alternative temperatures and durations of CMCT and alkaline treatment. We found that a 1-h CMCT treatment at 37°C on non-fragmented RNA combined with the 6 h of alkaline hydrolysis at 37°C resulted in the highest termination fold change (>5) at the expected position in the spike-in and significantly reduced noise compared with a variety of other treatment conditions that we attempted (Supplementary Fig. 1). We thus implemented this optimized CMCT treatment conditions for our Psi-seq library preparation.

Specifically, pelleted polyA+ RNA was resuspended in 30 μl 200 mM CMCT in BEU buffer (50 mM bicine, pH 8.3, 4 mM EDTA, and 7 M urea) or in 30 μl BEU buffer only (for mock samples) at 37°C on a thermomixer with 300 rpm rotation for 1 h. The CMCT reaction was stopped with 100 μl of 0.3 M sodium acetate and 0.1 mM EDTA (pH 5.6). After washing, the pellet was dried, dissolved in 40 μl of 50 mM sodium bicarbonate (pH 10.4) at 37°C for 6 h. Mock samples were handled identically in all steps but without CMCT treatment. RNA was then precipitated, washed, and dissolve in RNase-free water for library preparation.

Illumina sequencing libraries were prepared following the RNA ligation method as detailed (Schwartz *et al*. 2014). The single-stranded cDNA product was amplified for 9–11 cycles in a PCR. Libraries were pooled and sequenced on Illumina NextSeq 550 with high output of 75 bp paired end reads.

### Read trimming and mapping

Reads were adapter trimmed and quality filtered using Trimmomatic version 0.33 (Bolger *et al*. 2014) setting the cutoff threshold for average base quality score at 20 over a window of 3 bases, excluding the reads shorter than 20 bases post-trimming (parameters: leading = 20, trailing = 20, sliding window = 3, Minilen = 20). Alignment of the reads to the reference sequence was done in 2 phases. Cleaned reads were first mapped to a sequence reference comprising of the *D. melanogaster* 18S, 5.8S, 2S, and 28S ribosomal RNA gene sequences (accession: M21017.1) and the sequence for RNA spike-in (Schwartz *et al*. 2014) using bowtie2 (Langmead and Salzberg 2012). The remaining unmapped read pairs were extracted and mapped to the *D. melanogaster* genome reference release FB2017_04 (Larkin *et al*. 2021) using a splice-aware aligner, STAR version 2.6.1a (Dobin *et al*. 2013).

### Calculation of termination ratios and termination fold change

Inserts were identified by interpolating the concordantly mapped read pairs. For each insert, the 5′ end of the read R2 was identified as the termination site of the product from reverse transcription. For each sample, a termination ratio (TR) was computed as the ratio of number of reads terminating at the termination site to the total coverage at the site. Also, a termination fold change is computed for each site as the log2 ratio of TR in the CMC-treated sample (TR_CMC_) to that in the corresponding mock-treated sample (TR_Mock_).

### Identification of putative Psi sites

The termination sites with the TR_CMC_ being significantly larger than the corresponding TR_Mock_ were identified using a 2-proportion *z*-test.

Two proportion *z*-test at a given nucleotide position


$$H_{o}:\;TR_{\mathrm{CMC}}\leq TR_{\mathrm{Mock}}$$

$$H_{a}:\;TR_{\mathrm{CMC}}>TR_{\mathrm{Mock}}$$

Test statistic *z* is calculated as


$$z=\frac{TR_{\mathrm{CMC}}-TR_{\mathrm{Mock}}}{\sqrt{\left(\;p\;(1-p)\left(\frac{1}{\mathrm{Cv}g_{\mathrm{CMC}}}-\frac{1}{\mathrm{Cv}g_{\mathrm{Mock}}}\right)\right)}}$$

where Cvg_CMC_ and Cvg_Mock_ are the read coverage values in the CMC- and mock-treated samples, respectively, at the given nucleotide position and *p* is the pooled sample proportion which is calculated as


$$p=\frac{\left(\left(\mathrm{Cv}g_{\mathrm{CMC}}\;TR_{\mathrm{CMC}})+(\mathrm{Cv}g_{\mathrm{Mock}}\;TR_{\mathrm{Mock}}\right)\right)}{\left(\mathrm{Cv}g_{\mathrm{CMC}}+\mathrm{Cv}g_{\mathrm{Mock}}\right)}$$

The test is considered relevant only when there are at least 5 CMC-treated reads terminating at the position and another 5 continuing over the position.


$$(\mathrm{Cv}g_{\mathrm{CMC}}\times TR_{\mathrm{CMC}})\geq 5\;\;\mathrm{and}\;(\mathrm{Cv}g_{\mathrm{CMC}}\times \;(1-TR_{\mathrm{CMC}}))\geq 5$$

The *P*-values associated with the test static *z* (*z*-score) were adjusted for multiple testing using the Benjamini–Hochberg method. Termination sites from within the annotated gene regions with uridine as the upstream base and the adjusted *P*-value <0.05 in at least 2 *w^1118^* replicates, each from different experiments were considered potential Psi sites. Sites with significant adjusted *P*-values in 6 or 7 *w^1118^* replicates were categorized as high reproducibility sites, those in 4 or 5 *w^1118^* replicates as intermediate reproducibility sites and in 2 or 3 as low reproducibility sites.

### Calculation of expected number and *P*-value of Psi-containing codons on nuclear and mitochondrial mRNAs

The probability of occurrence for a given codon with pseudouridine replacing uridine at a given position was estimated as the ratio of the number of occurrences of that codon in the coding regions of the nuclear or mitochondrial mRNA genes (with at least 10× coverage in the *w^1118^* sample) to the total number of uridine-containing codons from those regions divided by the number of uridines within the codon. An expected number for each Psi-associated codon was estimated by multiplying its probability of occurrence with the total number of Psi sites associated with the coding regions (907 sites associated with nuclear mRNAs and 99 sites with mitochondrial mRNAs). *P*-values indicate the probability of finding more than the observed number of Psi sites by chance if the observed number was greater than the expected number or the probability of finding at most the observed number of Psi sites if the observed number was less than or equal to the expected number as calculated assuming binomial distribution.

### Identification of RluA-2-dependent sites

Psi sites called using our criteria described above in *RluA-2* and *w^1118^* of Exp 2 were compared. The Psi sites that were called in at least 2 *w^1118^* libraries from both of the experiments (Exp 1 and Exp 2) but not detected in any of the *RluA-2* mutant libraries are RluA-2 dependent and identified as RluA-2 targets.

### Detection of prevalent sequence motifs

For each RluA-2-dependent Psi site, a flanking sequence of 21 bp was extracted and a consensus was drawn using seqLogo version 2.8 (Crooks *et al*. 2004; parameters: -B 2 -T 0.25 -C 21 -k 1 -s -10 -c -n -Y).

### Differential gene expression analysis

Mock libraries of *RluA-2* were compared against *w^1118^* mock libraries to identify any significant differential gene expression. Read pairs mapping concordantly and uniquely to the exon regions of the annotated genes were counted using featureCounts tool ver. 2.0.0 of Subread package (Liao *et al*. 2014). Read alignments to antisense strand, or to multiple regions on the genome or those overlapping with multiple genes were ignored (parameters: -s 2 -p -B -C). Differential expression analysis was performed using DESeq2 ver. 1.12.3 (Love *et al*. 2014) and the *P*-values were corrected for multiple testing using the Benjamini–Hochberg method. Genes with adjusted *P*-values <0.05 were considered significantly differentially expressed.

### Gene set enrichment analysis

Gene set enrichment analysis (GSEA) was performed on a group of predefined gene sets derived from 137 Kyoto Encyclopedia of Genes and Genome pathways pertaining to *D. melanogaster* (Kanehisa and Goto 2000) to detect any significant, concordant differences between *RluA-2* and *w^1118^* gene expressions. The analysis was conducted using the software GSEA from Broad Institute (Subramanian *et al*. 2005) for which a matrix of DESeq2 normalized read counts for each gene were supplied as input (parameters: permute = gene set, metric = Signal2Noise, scoring_scheme = weighted).

## Results

### Optimization of Psi-seq library preparation conditions for efficient detection of putative pseudouridine sites

**Fig. 1.**
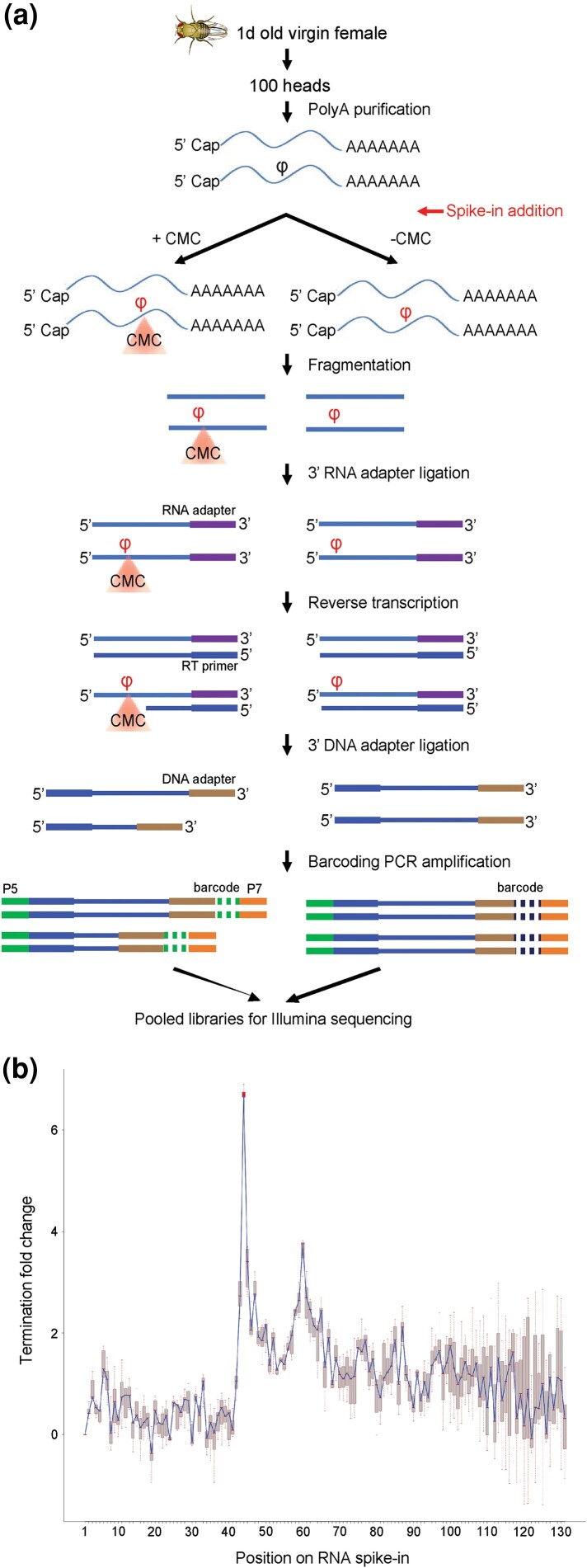
a) Schematic of *D. melanogaster* Psi-seq library construction. RNA was extracted from heads of unmated female flies. An in vitro transcribed RNA spike-in containing a single pseudouridine at position 43 was added into the RNA samples immediately after polyA selection and the optimized CMCT derivatization step (Supplementary Fig. 1) was carried out prior to RNA fragmentation and subsequent library preparation. Libraries were barcoded and pooled for the final Illumina sequencing. b) A connected box plot showing the termination fold change (log_2_) on each of the nucleic acid position along the synthetic RNA spike-in added to the genetic background *w^1118^* libraries (*n* = 7) used for pseudouridine mapping. With the optimized CMCT treatment (Supplementary Fig. 1), the termination fold change peak at the only Psi (position 43) of the RNA spike-in (highlighted with the red box plot) stands out among all the other positions.

We began our experiments with optimization of a previously used strategy (Schwartz *et al*. 2014) and applied the optimized technique to *Drosophila* head RNA. As an internal control to monitor, the effectiveness of the treatment and efficiency of the Psi detection, we spiked in a 150-bp in vitro transcribed RNA oligonucleotide containing a single pseudouridine at position 43 (Schwartz *et al*. 2014) at an early stage of library preparation. In pilot experiments, we identified experimental treatment conditions that allowed for robust calling of the pseudouridine of the RNA spike-in (Supplementary Fig. 1, details in Materials and methods). Our RNA samples were split into 2 equal halves immediately after polyA enrichment with one half subjected to CMCT treatment and the other to a mock treatment. This was followed by a ligation of an RNA adapter, an adapter-specific RT reaction, then a second ligation of a DNA adapter. The samples were then barcoded, amplified, and pooled for Illumina sequencing (Fig. 1a).

The RNA samples we used for the mapping of pseudouridylation were isolated from the heads of 1 day old virgin female flies of an iso*w^1118^* strain. This genetic background was previously used for behavioral characterization of *RluA-1* and *RluA-2* mutants (Song *et al*. 2020) and we focused on the head tissue since it would be enriched for genes expressed in the nervous system which are of greatest interest to us given the nociception phenotypes in the mutants. To assess repeatability, we carried out 2 sets of experiments. In Exp 1, we prepared, sequenced, and analyzed 3 pairs of *w^1118^* libraries (with each pair containing a CMCT and mock treatment). In Exp 2, we performed the procedures on 4 pairs of *w^1118^* and 4 pairs of *RluA-2^del-FRT^* mutant (Supplementary Table 1 in File 1). An average of 91% of the paired reads from the 22 libraries had both mates cleared through quality filters and >87% of the reads could be mapped concordantly (Supplementary Table 2 in File 1), confirming the high quality of the sequencing reads for the libraries from both experiments.

To call potential Psi sites, we determined a *termination ratio* for all nucleotide sites in the sequenced libraries. The termination ratio was computed as the ratio of read counts that terminated at a specific site in CMC-treated samples and the total coverage in mock-treated samples. Since the CMC treatment blocks reverse transcription adjacent to a Psi site, a higher termination ratio in the CMC library compared with the corresponding mock library is an indicator of the location of a potential Psi residue. We then used the log2 of termination ratio in the paired CMC and mock libraries to define *termination fold change*. Our bioinformatics pipeline, when applied to the synthetic spike-in RNA in our experiments resulted in termination fold change at the expected position adjacent to the Psi site that was >6 across all pairs of CMC and mock libraries (Supplementary Table 2 in File 1). A termination fold change >6 represents an average absolute fold change >64 (since log_2_(64) = 6; Fig. 1b). This result for the RNA spike-in indicates a highly efficient CMCT treatment which produced strong termination adjacent to the Psi site.

### Pseudouridylation of *Drosophila* ribosomal RNAs

Previous studies have mostly employed subjectively chosen thresholds for Psi site calling, i.e. a combination of “Psi ratio” (equivalent to our “termination ratio”) > 0.1 and “Psi fold change” (equivalent to our “termination fold change”) of >3 were used to identify the candidate pseudouridine sites in yeast and human cell lines (Schwartz *et al*. 2014). We found that applying the same thresholds to our data resulted in the calling of an extremely high number of sites. For instance, if we applied the thresholds of Schwartz *et al* (2014), we identified over 200,000 sites across 7 *w^1118^* libraries. In addition, we found that reproducibility of sites called using this thresholding method was low when comparing across replicates. The very high number of sites using the thresholding and the lack of reproducibility across samples caused us to question the robustness of this calling method for our experimental conditions.

To overcome this limitation, we analyzed the potential Psi sites according to the statistics of the termination ratios that we observed. To do this, we applied a 2-proportion *z*-test to evaluate whether the termination ratio at a specific site in the CMC-treated sample was significantly higher than in the mocked treated sample. Sites with an associated False Discovery Rate (FDR) < 0.05 are statistically more likely to terminate than expected and were called as potential Psi sites. In addition, we expect biologically meaningful sites to show reproducibility across samples, thus we required that a potential Psi site was called in at least 2 *w^1118^* replicates across the 2 independent experiments (details in Materials and methods). Finally, we categorized the Psi sites according to high reproducibility (identified in 6–7 paired replicates), intermediate reproducibility (identified in 4–5 paired replicates), or low reproducibility (identified in 2–3 paired replicates).

**Fig. 2.**
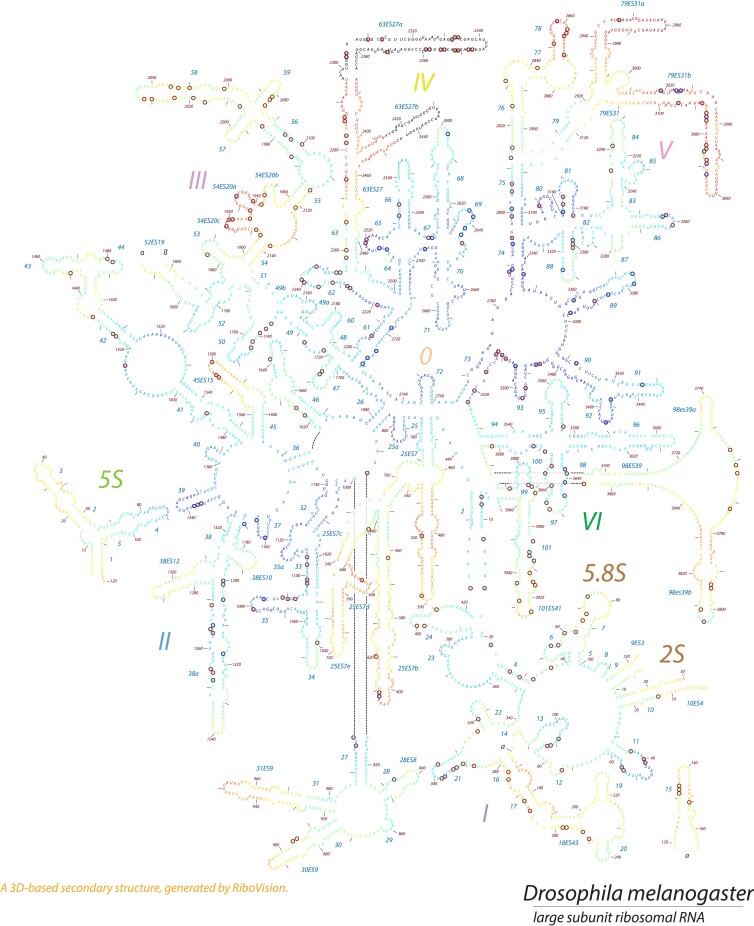
Location of pseudouridine modification sites (circled U) on cytoplasmic LSU rRNA secondary structure in *D. melanogaster*. Psi residues are circled in red for high reproducible sites and in blue for previously identified Ofengand sites. The residues are color coded according to the “fine grained onion” scheme in which close proximity to the peptidyl transferase center is indicated by cooler colors (i.e. blue) and sites far from the center are indicated by warmer colors.

According to these criteria, we identified 735 potential Psi sites on cellular rRNA which survived the polyA enrichment of our samples (Supplementary Table 3 in File 1). Although this level of Psi on rRNA vastly exceeds the number of previously identified sites, 532 of the sites (72.4%) were highly reproducible. These highly reproducible sites on cellular rRNA are distributed across rRNA classes including 288 Psi sites in 28S, 16 sites in 5.8S, as well as 228 sites in 18S rRNAs (Supplementary Table 3 in File 1). In *D. melanogaster*, only 57 Psi sites on the large subunit (LSU) of cytoplasmic rRNA have been previously reported using the canonical primer-extension-based pseudouridine detection method (Ofengand and Bakin 1997; “Ofengand site” indicated by gray vertical lines in Supplementary Fig. 2). Using our method of Psi calling, we have identified 53 of those known LSU sites (labeled as Ofengand site in Column H in Supplementary Table 3 in File 1). Notably, 49/53 of those known sites are highly reproducible Psi sites in our analysis (Supplementary Table 3 in File 1). In Fig. 2, the cytoplasmic rRNA Psi sites are mapped onto the 3D-based secondary structure of LSU rRNA of *D. melanogaster* (RiboVision version 1.15+; Bernier *et al*. 2014). As there is no obvious clustering of the sites, additional analyses will be required to understand how the patterns observed relate to ribosomal function.

In addition to the 735 potential Psi sites identified on cytoplasmic rRNA, we also found 125 sites on mitochondrial rRNA, among which 57 highly reproducible sites were identified on the mitochondrial large ribosomal RNA (lrRNA; Supplementary Table 3 in File 1). Previously studies have identified a single pseudouridylation site in human mitochondrial 16S rRNA (Psi1397) which is essential for its stability and assembly into the mitochondrial ribosomes (Antonicka *et al*. 2017). Our results suggest a more widespread occurrence of Psi sites on mitochondrial rRNA in *D. melanogaster*.

### Psi-seq identified predicted and novel Psi in snRNAs, snoRNAs, tRNAs, and other ncRNAs

Like the ribosome, the spliceosome is an important catalytic ribonucleoprotein machine. Numerous Psi sites have been reported in the spliceosomal snRNAs U1, U2, U4, U5, U6, U12, U4atac, and U6atac (Yu *et al*. 2011; Adachi and Yu 2014). Although our protocol relied on polyA enriched RNA, we identified potential pseudouridine modifications on 4 spliceosomal snRNAs, including 21 on U1, 11 on U2, 2 on U5, and 5 on U6 (Supplementary Table 4 in File 1). Six of these (Psi5, Psi6, Psi22 on U1, Psi44, Psi45, Psi55 on U2) were previously reported or predicted in *Drosophila* snRNAs (Huang *et al*. 2005; Deryusheva and Gall 2009). Additional sites (i.e. Psi46 on U5 and Psi40 on U6) were reported at the corresponding locations in human (Karijolich and Yu 2010) but have not been previously identified in *Drosophila* spliceosomal snRNA.

7SK is another important and highly conserved snRNA. 7SK is pseudouridylated at position U250 by the DKC1-box H/ACA RNP in human and this pseudouridylation was shown to be critical to stabilize 7SK snRNP and its function as a critical regulator of the homeostasis and activity of P-TEFb, a key regulator of RNA polymerase (pol) II transcription which stimulates the elongation phase (Zhao *et al*. 2016). This modification was identified from previous Psi-seq study (Carlile *et al*. 2014). The 7SK snRNA homolog and a similar P-TEFb control system function in *Drosophila* (Gruber *et al*. 2008; Nguyen *et al*. 2012). Our pseudo-seq analysis identified a single nucleotide pseudouridylation modification in d7SK snRNA, at U269 (Supplementary Table 4 in File 1).

We found 10 Psi sites on the U3 RNA, a box C/D snoRNA required for rRNA processing. All the sites are distinct from the 4 Psi sites found on human U3 using mass spectrometry (Yamaki *et al*. 2020). There is also a single Psi site identified on snoRNA:Psi 28S-3342. Another 58 sites are localized on other ncRNAs, including 47 on 7SL, an abundant cytoplasmic RNA which functions in protein secretion as a component of the signal recognition particle. Previously only 1 Psi site (Psi211) has been reported on human 7SL. There are 11 sites on CR40469, an uncharacterized lncRNAs (Supplementary Table 4 in File 1).

Our data indicate 8 Psi sites in *Drosophila* tRNAs (Supplementary Table 4 in File 1), 3 of which (Psi13 and Psi54 of Glu-TTC and Psi27 of Val-CAC) were previously mapped on tRNAs of *D. melanogaster* (Machnicka *et al*. 2014).

All the Psi sites found on the ncRNAs, except for those on cytoplasmic and mitochondrial rRNAs in Supplementary Table 3 in File 1, are listed in Supplementary Table 4 in File 1 and can be sorted by the RNA biotype (column F). Note that more ncRNA sites are likely to be identified using targeted approaches that do not rely on polyA enrichment.

### Psi modifications of mRNA transcripts

**Fig. 3.**
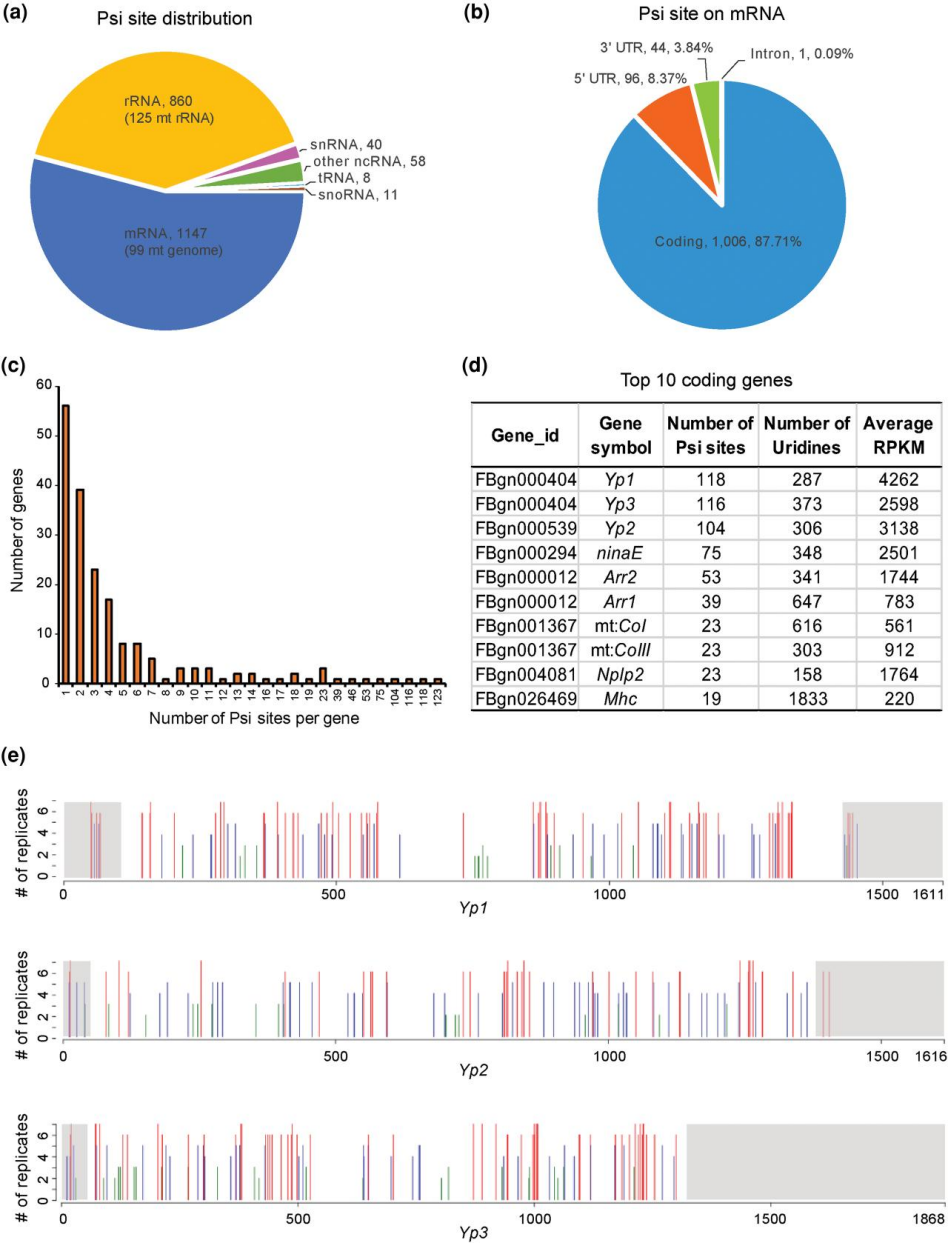
Features of pseudouridine sites identified from *D. melanogaster* transcriptome. a) Psi site distribution according to RNA class (mRNA, rRNA, tRNA, snRNA, snoRNA, and other ncRNA). b) Distribution of Psi sites on mRNA transcript regions (5′-UTR, coding, 3′-UTR, and intron). c) Distribution of number of Psi sites identified per gene. d) The 10 coding genes with the highest number of Psi sites. For each gene, the numbers of Psi sites and uridines in the sequence are listed along with the normalized gene expression represented as average RPKM of the *w^1118^* CMC libraries (*n* = 7). e) Psi sites on yolk protein mRNAs. The locations of Psi sites identified on mRNAs for yolk protein *Yp1*, *Yp2*, and *Yp3* are indicated with bars above the schematic of the transcripts. UTRs are marked with gray shading whereas coding regions are not shaded. The height of the bars indicated by the *y*-axis represents the number of *w^1118^* replicates a Psi site is identified from (*n* = 2–7) while red, blue, and green color of the lines represents Psi sites of high, intermediate, and low reproducibility, respectively.

It was previously unknown if Psi occurs on *Drosophila* mRNAs. Our analysis identified 1,147 mRNA Psi sites including 287 highly reproducible sites, 489 sites with intermediate reproducibility, and 371 with low reproducibility (Fig. 3a; Supplementary Table 5 in File 1). Interestingly, a large proportion of pseudouridylated mRNAs have multiple sites identified in their transcripts (Fig. 3c). Indeed, the 1,147 Psi sites are localized on the transcripts of only 165 genes with transcripts from some loci containing >100 sites (Fig. 3, c and d; Supplementary Table 5 in File 1).

In *Drosophila*, the ribosomal LSU (RpL, 60S) consists of 3 rRNAs (2S, 5.8S, 28S) and 47 ribosomal proteins whereas the small ribosomal subunit (RpS, 40S) consists of 1 ribosomal RNA (18S) and 33 ribosomal proteins (Anger *et al*. 2013). Our analysis identified 202 Psi sites in mRNAs encoding 35 of 47 RpL proteins and 23 of 33 RpS proteins. As with other mRNAs, most mRNAs encoding RPs contain at least 2 Psi sites. Although the vast majority the ribosomal proteins are pseudouridylated, we found no Psi modifications on the mitochondrial ribosomal protein mRNAs even though these were abundantly expressed in our RNA samples. Altogether, the 202 Psi sites on ribosomal protein RNAs accounted for 17.6% of total pseudouridines identified on mRNAs. In addition, if adding together the 735 Psi sites on the cytoplasmic rRNAs and 202 sites on various ribosomal protein mRNAs, nearly 44% of the Psi sites that we have identified are related to the production of cytoplasmic ribosomes.

Among the mRNA Psi sites, a total of 99 Psi sites were found on protein-coding genes of the mitochondrial genome (Fig. 3a; Supplementary Table 5 in File 1). Eleven of the 13 protein encoding mRNAs in the mitochondrial genome were pseudouridylated. Considering the 125 Psi sites found on the mitochondrial rRNA (Supplementary Table 3 in File 1), we found a total of 224 Psi sites on the RNAs encoded from mitochondrial genome (10.5% of the total sites), thus indicating that RNAs from mitochondrial genome are significantly pseudouridylated in *Drosophila*.

As noted above, many of the mRNA transcripts have multiple Psi sites. Interestingly, Psi sites are found across functionally related mRNA transcripts. For instance, the mRNAs encoding the yolk proteins, which are known to be highly expressed in the fat bodies of adult female head (Fujii and Amrein 2002), contain the highest number of pseudouridine modification of the female head transcriptome. A total of 338 Psi sites (25.5% of total mRNA Psi sites) were identified on mRNAs encoding the 3 yolk proteins (Yp1, Yp2, and Yp3). Each of the yolk protein mRNAs harbors many Psi sites (118 sites on *Yp1*, 104 on *Yp2*, and 116 sites on *Yp3*; Fig. 3e; Supplementary Table 5 in File 1). Notably, the majority of the Psi sites are localized in the coding region and are almost absent from the 3′-untranslated region (UTR) region of the transcripts, which confirms the specificity of our Psi-seq analysis and strongly suggests for an important function of pseudouridylation in the coding region for the yolk protein transcripts.

Transcripts of multiple genes involved in sensory function in the nervous system are also marked with Psi modifications. A notable example is *ninaE*, the structural gene encoding the *Drosophila* Rhodopsin-1, with 75 Psi sites. Indeed, a number of transcripts bearing multiple Psi sites play various roles in photoreceptor function, including other *rhodopsins* (*Rh*) (*Rh3*: 2 Psi sites, *Rh4*: 2 Psi sites, *Rh6*: 2 Psi sites), *retinin* (18 Psi sites), and *Arrestins* (*Arr1*: 39 Psi sites, *Arr2*: 53 Psi sites). Several *odorant-binding protein* mRNAs, which function in the sensory perception of smell, also have multiple Psi sites (*Obp19d*: 3 Psi sites, *Obp44a*: 12, *Obp56d*: 3, *Obp56e*: 2, *Obp99c*: 1). It is interesting that mRNAs encoding 2 potential neuropeptide genes *Nplp2* and *Nplp3* contain 28 and 23 Psi sites, respectively (Supplementary Table 5 in File 1).

Previously, an N3-CMC-enriched pseudouridine sequencing identified pseudouridylation at U519 of the human elongation factor *eEF1A1* mRNA which encodes the main protein that delivers aminoacyl-tRNAs to the ribosome. In fact, *eEF1A1* is the first experimentally validated human mRNA psi site (Li *et al*. 2015). From our Psi-seq data, sites were identified on mRNA for two of the *Drosophila* translation elongation factors (*eEF1α1* with 4 sites and *eEF1γ* with 1 site; Supplementary Table 5 in File 1).

The majority of Psi sites (1,006 sites, 87.7%) found on mRNAs were within the coding region. The 5′-UTRs had only 98 (8.5%) sites and 61 sites (5.3%) were in 3′-UTRs (Fig. 3b). In coding regions, Psi sites were more commonly found in the second position of the codon relative to the first or third positions. For nuclear-encoded mRNA, the most highly pseudouridylated codons are the CΨG and UΨG for leucine, followed by GCΨ for alanine and ΨGG for tryptophan (Table 1). Interestingly, the pattern of pseudouridylation of codons in mitochondrial genome transcripts appeared to be distinct from that of nuclear genome transcripts, with the most frequent Psi-containing codons encoding for phenylalanine (F) (i.e. UUΨ and UΨU), isoleucine (I) (i.e. AΨU), as well as leucine (but in the mitochondrial case, ΨUA was the most prominent codon for leucine; Table 1).

**Table 1.** Distribution of Psi (Ψ) sites on nuclear or mitochondrial genome-encoded codons and amino acids in *D. melanogaster*.

	Encoded by nuclear genome*^a^*					Encoded by mitochondrial genome*^b^*					
Codon*^c^*	Amino acid*^d^*	Abundance*^e^*	Expected Count*^f^*	Psi count*^g^*	Adj. pval*^h^*	Codon*^c^*	Amino acid*^d^*	Abundance*^e^*	Expected Count*^f^*	Psi count*^g^*	Adj. pval*^h^*
ΨAA	STOP	5,749	1	0	1.00	ΨAA	STOP	9	0	0	1.00
ΨAG		4,827	1	0	1.00	ΨAG		1	0	0	1.00
ΨGA		3,321	1	0	1.00	ΨGA	W	68	3	5	1.00
**GCΨ**	**A**	107,656	26	45	1.29E−02^a^	GCΨ	A	88	4	3	1.00
ΨGC	C	99,092	24	21	1.00	ΨGC	C	1	0	0	1.00
ΨGU		43,290	5	9	1.00	ΨGU		20	0	1	1.00
UGΨ		43,290	5	2	1.00	UGΨ		20	0	0	1.00
**GAΨ**	**D**	207,714	51	23	2.93E−04^c^	GAΨ	D	36	2	0	1.00
ΨUC	**F**	160,233	20	31	2.77E−01	ΨUC	**F**	13	0	0	1.00
UΨC		160,233	20	14	1.00	UΨC		13	0	0	1.00
ΨUU		105,347	9	10	1.00	ΨUU		219	4	3	1.00
UΨU		105,347	9	12	1.00	UΨU		219	4	9	1.73E−01
UUΨ		105,347	9	11	1.00	**UUΨ**		219	4	12	3.51E−03^b^
GGΨ	**G**	96,329	24	22	1.00	GGΨ	G	28	1	1	1.00
CAΨ	**H**	79,960	20	14	1.00	CAΨ	H	51	3	0	1.00
**AΨA**	**I**	75,401	18	1	7.44E−06^c^	AΨA	I	129	6	0	6.13E−02
**AΨC**		164,918	40	7	2.99E−09^c^	AΨC		13	1	0	1.00
AΨU		127,100	16	21	1.00	AΨU		249	6	12	4.38E−01
**AUΨ**		127,100	16	30	1.54E−02^a^	AUΨ		249	6	8	1.00
**CΨA**	**L**	64,763	16	2	7.87E−04^b^	CΨA	L	18	1	0	1.00
**CΨC**		102,941	25	6	2.00E−04^c^	CΨC		0	0	0	1.00
**CΨG**		281,351	69	201	9.12E−42^c^	CΨG		0	0	0	1.00
CΨU		70,463	9	13	1.00	CΨU		22	1	0	1.00
CUΨ		70,463	9	10	1.00	CUΨ		22	1	1	1.00
ΨUA		36,665	4	1	1.00	ΨUA		335	8	12	1.00
UΨA		36,665	4	0	5.31E−01	UΨA		335	8	7	1.00
**ΨUG**		124,790	15	33	9.98E−04^b^	ΨUG		9	0	1	1.00
**UΨG**		124,790	15	46	1.65E−09^c^	UΨG		9	0	1	1.00
AΨG	**M**	174,201	43	48	1.00	AΨG	M	6	0	1	1.00
**AAΨ**	**N**	162,114	40	11	2.22E−06^c^	AAΨ	N	125	6	5	1.00
CCΨ	**P**	54,987	13	12	1.00	CCΨ	P	56	3	0	1.00
CGΨ	**R**	64,053	16	19	1.00	CGΨ	R	7	0	0	1.00
**AGΨ**	**S**	90,230	22	3	2.02E−05^c^	AGΨ	S	17	1	1	1.00
**ΨCA**		61,588	15	1	1.93E−04^c^	ΨCA		77	4	0	9.50E−01
**ΨCC**		144,883	36	12	1.65E−04^c^	ΨCC		6	0	0	1.00
**ΨCG**		120,743	30	9	3.59E−04^c^	ΨCG		3	0	0	1.00
ΨCU		54,711	7	4	1.00	ΨCU		74	2	3	1.00
UCΨ		54,711	7	9	1.00	UCΨ		74	2	1	1.00
ACΨ	**U**	76,117	19	8	2.15E−01	ACΨ	U	73	4	2	1.00
**GΨA**	**V**	49,291	12	0	2.52E−04^c^	GΨA	V	65	3	1	1.00
**GΨC**		100,296	25	8	4.15E−03^b^	GΨC		1	0	0	1.00
GΨG		204,607	50	68	2.55E−01	GΨG		6	0	0	1.00
**GΨU**		84,822	10	22	2.19E−02^a^	GΨU		52	1	2	1.00
GUΨ		84,822	10	18	4.81E−01	GUΨ		52	1	2	1.00
**ΨGG**	**W**	74,214	18	32	4.79E−02^a^	ΨGG	W	2	0	0	1.00
ΨAC	Y	136,903	34	23	1.00	ΨAC	Y	16	1	0	1.00
ΨAU		85,677	10	6	1.00	ΨAU		87	2	0	1.00
UAΨ		85,677	10	9	1.00	UAΨ		87	2	5	1.00

Distribution of Psi in codons of the transcripts encoded by nuclear genome of *D. melanogaster*.

Distribution of Psi in codons of the transcripts encoded by mitochondrial genome of *D. melanogaster*.

Codon: Codon with one of the uridines substituted with Psi site. The codons with significant Adj.pval are given in bold.

Amino acid: Product of translation. The amino acids/stop codons with significant Adj.pval (not shown) are given in bold.

Abundance: Count of the given codon in the transcriptome.

Expected count: An expectation for the count of Psi sites associated with the given codon is estimated from the observed count by multiplying the total number of Psi sites on mRNA with the ratio of abundance of the given codon divided by the count of uridines to the total count of codons in the transcriptome.

Psi count: Count of the Psi sites overlapping with the given codon. When multiple transcripts of a given gene are involved, the codon from the longest transcript is considered.

Adj_pval: adjusted *P*-value. Assuming a binomial distribution for the observed Psi count, a *P*-value indicating the cumulative probability of the count of Psi sites associated with the given codon being at least the observed count if found greater than the expected count (or at most the observed count if found less than the expected count) was calculated. The *P*-values are then corrected for multiple testing using Bonferroni's method.

^a^ *P* < 0.05. ^b^*P* < 0.01. ^c^*P* < 0.001.

### Pseudouridylation of the ochre UGA codon in *Drosophila* mitochondria

A previous study proposed that Psi in mRNA stop codons may result in the suppression of translation termination and enhancement of translational read-through *in vitro* protein synthesis (Karijolich and Yu 2011). We were very interested in this as a set of transcripts in the *Drosophila* transcriptome are predicted to undergo stop-codon suppression (Jungreis *et al*. 2011). However, our analysis failed to identify any Psi sites on stop codons of nuclear-encoded genes (Table 1; Supplementary Table 5 in File 1). This result suggests that pseudouridylation is unlikely to be an important underlying mechanism for stop-codon readthrough in *Drosophila*.

However, we did find Psi sites present on the canonical “stop-codon” UGA in mitochondrial encoded transcripts. Out of the 68 total UGAs in mitochondrial mRNA, 5 were called as ΨGA from our analysis. This was in contrast to 3,321 UGAs that were present among the nuclear mRNA that did not contain a signal for ΨGA. The frequency of ΨGA codons in mitochondrial RNA was significantly higher than in nuclear-encoded RNA (Fisher's exact test *P* = 2.81e–9). Importantly, the UGA codon in mitochondria of insects and in vertebrates does not encode a stop, but instead codes for tryptophan. Our analysis suggests that this codon, at least in some cases, is actually ΨGA.

### mRNA pseudouridylation is correlated with abundance

**Fig. 4.**
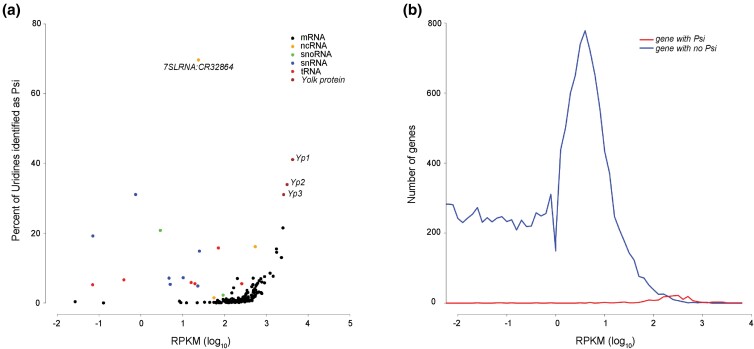
Correlation between gene expression and Psi site identification. a) A scatter plot of gene expression vs proportion of Psi site across pseudouridylated transcripts. The gene expression is represented as the average of log_10_ (RPKM) in *w^1118^* CMC libraries (*n* = 7) and the proportion of Psi sites is measured by the percentage of Psi sites called on the total number of uridines in various categories of RNA species. The individual data points representing the yolk proteins and 7SL RNA are labeled. b) A plot of gene expression vs the number of genes with (red) or without (blue) Psi sites identified on their transcripts.

The yolk protein mRNAs are among the most highly expressed transcripts in our female fly head libraries and these were the most highly pseudouridylated genes. mRNAs encoding ribosomal proteins are also abundantly expressed and were among the classes of genes with a high likelihood of Psi modification in their transcripts. Thus, we explored the relationship between Psi occurrence and RNA expression level in CMC library (Fig. 4a). Interestingly, for mRNAs there appeared to be a strong positive correlation between mRNA expression level and the number of Psi sites in a particular transcript. The majority of the Psi sites are identified on the transcripts of the genes which are abundantly expressed (Fig. 4b). However, this was unlikely to be caused by a limitation coverage in calling sites because we found no correlation between the read coverage and the presence of Psi sites for the ncRNAs, such as snRNA, snoRNA, tRNA, and other ncRNA (Fig. 4a). Our results do not allow us to infer a cause and effect for the frequency of Psi on *Drosophila* mRNAs. Highly expressed mRNAs may be more stable due to the presence of Psi modification, or they may be more likely to be pseudouridylated because they are more highly expressed, or both factors may contribute to varying degrees.

### Transcriptome-wide identification of potential pseudouridylation targets of RluA-2

**Fig. 5.**
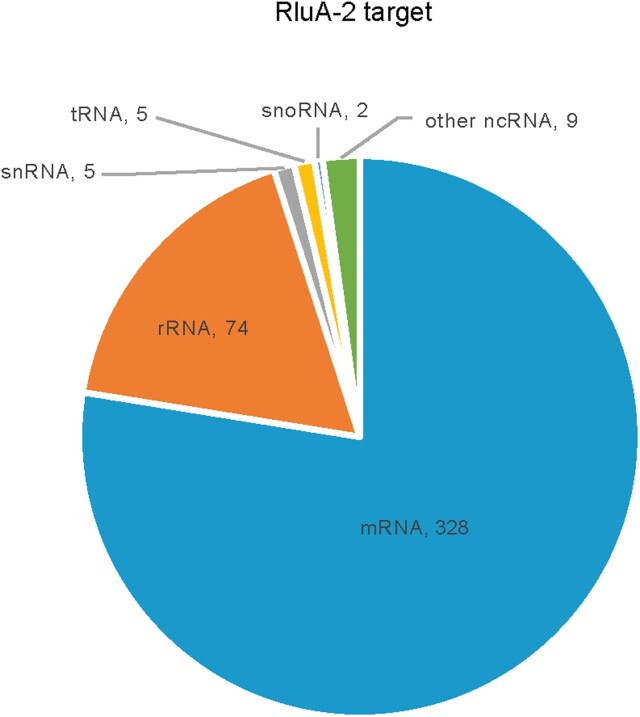
Pie chart showing the distribution of number of RluA-2-dependent Psi sites in different categories of RNA species (mRNA, rRNA, tRNA, snRNA, snoRNA, and other ncRNA). Those potential RluA-2 target sites are missing from *RluA-2* mutant compared with the *w^1118^*.

Previously, the putative pseudouridine synthase RluA-2 was found to be broadly expressed in *Drosophila* and loss of function mutation in *RluA-2* was found to cause hypersensitivity in larval pain pathways (Song *et al*. 2020). Thus, we sought to identify potential targets for RluA-2 by comparing the pseudouridine profiles of *RluA-2* mutant mRNA with *w^1118^* control mRNA. A total of 423 Psi sites were not detected in any of the 4 *RluA-2* mutant libraries. These missing sites therefore represent potential targets of RluA-2 (Supplementary Table 6 in File 1). The candidate RluA-2 target sites include 74 pseudouridylation sites on cytoplasmic and mitochondrial rRNAs (63 cellular and 11 mitochondrial), 5 on snRNAs, 5 on tRNA, 9 on other ncRNAs, and 328 sites on mRNAs (Fig. 5). The mRNA Psi sites include 65 of the sites encoding for components of ribosomal proteins and 42 mitochondrial genome-encoded transcripts. Among the 165 genes where the pseudouridylation sites were identified on *w^1118^* transcripts (Supplementary Table 5 in File 1), 115 genes were missing Psi modifications in the *RluA-2* mutant. That the sites were missing in various classes of RNA suggests that the RluA-2 enzyme acts on a wide range of substrates (Supplementary Table 6 in File 1).

To determine if RluA-2 recognizes a particular sequence motif, we ran the target sites through a motif finding algorithm. Other than a dominant G downstream of the pseudouridine site, there was not a significant sequence logo around the modification site (Supplementary Fig. 3). This finding suggests that RluA-2 enzyme may not recognize a primary sequence motif in site selectivity and may indicate that secondary structure of RNAs is critical for RluA-2 target recognition (as has been found for other pseudouridine synthases; Purchal *et al*. 2022).

### Loss of RluA-2 results in differential expression in translation-related genes and ncRNAs

**Fig. 6.**
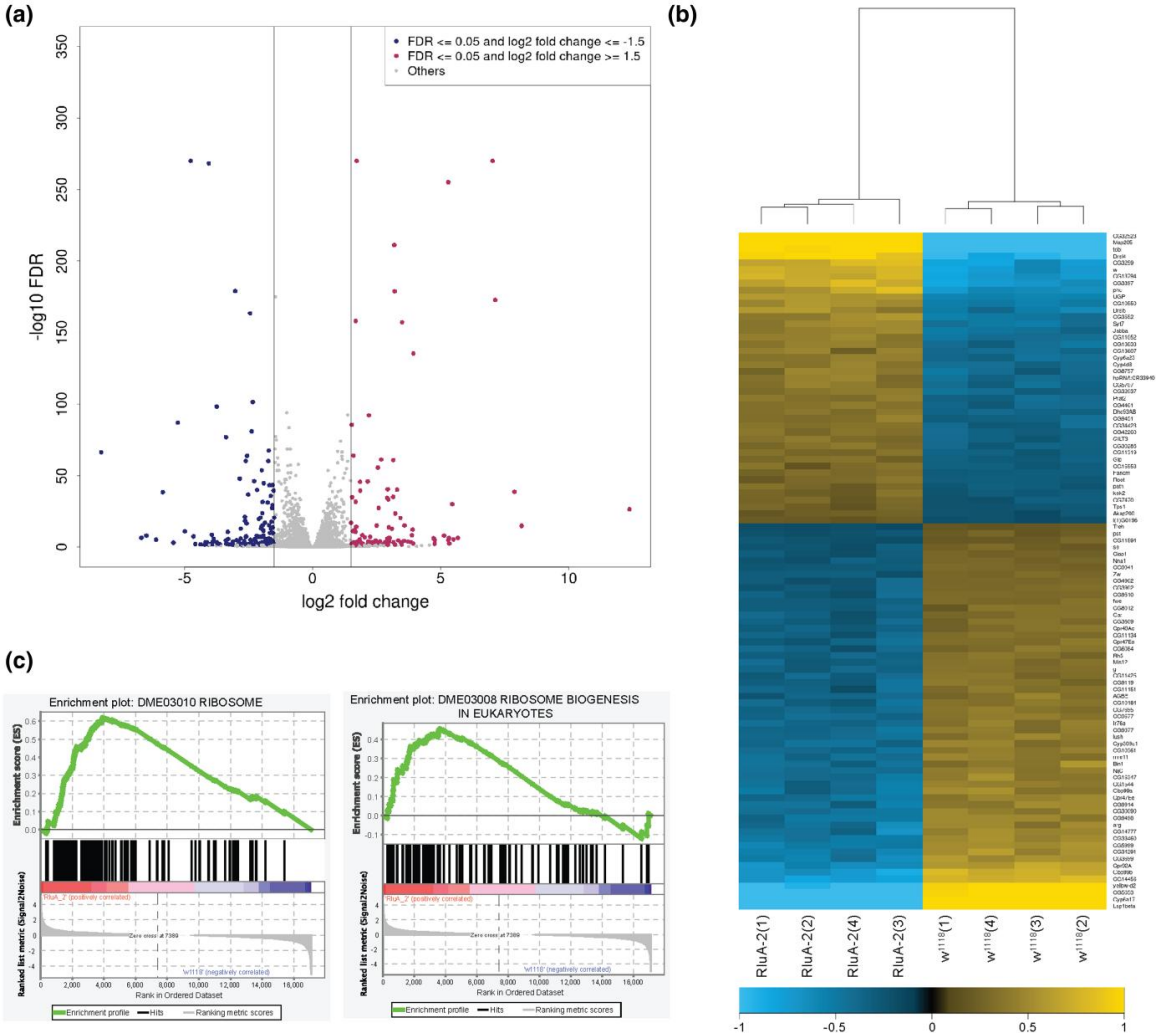
Effect of loss of RluA-2 activity on gene expression in the female *Drosophila* head transcriptome. a) Volcano plot showing the differentially expressed genes in the mock Psi-seq libraries of *RluA-2* vs *w^1118^*. The log_2_-fold change indicated the mean expression level for each gene. Each data point represents an individual gene. Red data points indicate upregulated genes with log_2_-fold change ≥1.5, the blue data points indicate downregulated genes with log_2_-fold change ≤1.5 in *RluA-2* vs *w^1118^* (FDR ≤ 0.05) and gray data points represent all the other genes. b) A hierarchically clustered heatmap showing the expression pattern of the top 100 significantly differentially expressed genes between *RluA-2* and *w^1118^*. Gene expression values quantified as the counts of reads mapping to the exon regions were transformed by applying DESeq2's regularized-logarithm transformation. The rows representing the genes were sorted in descending order of their average expression among the *RluA-2* libraries. The columns representing the individual libraries were hierarchically clustered based on the gene expression. The color density on the heatmap indicates the extent of deviation for each gene in each replicate library from the gene's average expression across all libraries. Yellow and blue represent up- and downregulated expression in *RluA-2*, respectively. c) Gene set enrichment plots show that the Gene Ontology categories are enriched for the ribosome (left) and ribosome biogenesis in eukaryotes (right) in the *RluA-2* mutants. The *y*-axis in the top portion represents enrichment score (ES) and on the *x*-axis are genes (vertical black lines) represented in genes sets. The green line connects points of ES and genes. ES is the maximum deviation from zero as calculated for each gene going down the ranked list, and represents the degree of over-representation of a gene set at the top or the bottom of the ranked gene list. The middle portion shows where the members of the gene set appear in the ranked list of genes. The colored band represents the degree of correlation of genes with *RluA-2* (red for positive and blue for negative correlation). The ranking metric at the bottom portion shows a gene's correlation with *RluA-2* (positive value) or *w^1118^* (negative value). The significantly enriched gene sets are DME03010 RIBOSOME (*P* = 0.000; FDR = 0.000) and DME03008 RIBOSOME BIOGENESIS IN EUKARYOTES (*P* = 0.000; FDR = 0.154).

To investigate the effects of *RluA-2* on gene expression, we compared the transcriptome of *RluA-2* mutant to the control genetic background iso*w^1118^* in mock-treated libraries of Exp 2. The Euclidean distance of the libraries demonstrated a clear clustering of samples based on genotype (Supplementary Fig. 4). A total of 2,214 genes showed significant change in their expression levels in the *RluA-2* vs iso*w^1118^* samples (FDR < 0.05), including 1,048 that were upregulated and 1,166 that were downregulated in the mutant (Fig. 6a; Supplementary Table 7 in File 1). The vast majority of differentially expressed genes (94.8%) were mRNA-coding genes, which included 994 of the upregulated and 1,104 of the downregulated genes. Among the upregulated gene set, we found 35 RpL genes and 22 RpS genes, 3 elongation factors and 11 initiation factors (Fig. 6b; Supplementary Table 7 in File 1). It is notable that *Drosophila* Myc, which is a major regulator of genes involved in ribosome biogenesis and translation (Gallant 2013), as well as its binding partner dMax, were both upregulated in *RluA-2*. Consistent with these observations, a GSEA of the differentially expressed genes showed significant enrichment in the ribosome (FDR < 0.0001) and ribosome biogenesis (FDR = 0.154; Fig. 6c). These results suggest a potential impact on the translation and translational machinery that is caused by loss of RluA-2.

Notably, 77 ncRNAs (non-protein-coding antisense RNAs and long ncRNAs) were either upregulated (47) or downregulated (30) and several snoRNAs responsible for guiding pseudouridylation or methylation on 18S and 28S rRNAs were among the differentially regulated RNAs (Supplementary Table 7 in File 1).

We investigated impact of loss of RluA-2 on the other 8 putative pseudouridine synthases. Expression of *RluA-1*, the paralog of *RluA-2*, was slightly decreased which may be a consequence of loss of regulatory DNA in the *RluA-2* deletion. In contrast, *mfl* the human *Dyskerin* homolog and *CG3709* (encoding the putative *Drosophila* Pus10) were upregulated although the fold change in expression was also slight (Supplementary Table 7 in File 1). The expression of the other putative pseudouridine synthases is either not changed (*CG3045*, *CG4159*/*PUS1*, and *CG34140*) or not detected in female head (*CG7849* and *CG6745/PUS7*).

Finally, we investigated whether there might be a correlation between RluA-2-dependent pseudouridylation and gene expression. If such a correlation exists, transcripts that lose sites in the mutant might show altered transcript levels. Interestingly, 67 genes with missing Psi sites in the *RluA-2* mutant also show differential expression (Supplementary Table 8 in File 1). Among the RluA-2 targets, ribosomal protein component genes are also differentially expressed (18/21 *RpL* and 9/15 *RpS* genes). The differentially expressed mRNAs encoding ribosomal proteins all showed upregulation in the *RluA-2* mutant. A similar trend of upregulation was also observed in other RluA-2 target genes, including yolk protein mRNAs (*Yp1*, *Yp2*, and *Yp3*). However, some transcripts missing Psi sites were found to be reduced in expression (i.e. *Nplp3, Obp19d,* and *Obp44a*). The vast majority of the differentially expressed genes did not contain RluA-2-dependent Psi sites (2,147, 97.0%), suggesting the majority of the differentially expressed genes do not change expression levels as a consequence of a missing pseudouridylation. This may indicate that *RluA-2* can directly affect RNA levels independently of its pseudouridine synthase activity, or the gene expression alterations may be a downstream or indirect consequence of a lack of *RluA-2* activity. Future studies investigating the RNAs that are directly bound by RluA-2 will be necessary to disentangle these alternatives.

## Discussion

For over a century studies of *D. melanogaster* have led to countless insights into the underlying principles of genetics and molecular biology including several studies of pseudouridylation of RNA. Nevertheless, next generation sequencing has yet to be applied to thoroughly characterize *Drosophila* Psi. We have described here the first attempt at characterizing a *D. melanogaster* pseudouridine epitranscriptome. Our optimized library construction and bioinformatics workflow has allowed us to robustly detect previously known sites as well as to uncover a set of previously unknown Psi sites that are reproducible across biological replicates. Although our analysis has been limited to a single stage of the fly life cycle, and a single sex, our findings provide interesting insights for future studies.

Perhaps the most striking finding of our study is the widespread presence of Psi in the RNAs that encode for the many components of the cellular ribosome. Over 500 highly reproducible sites were found in rRNA which is significantly more than the number of previously identified sites. The vastly increased number of candidate rRNA sites that we have detected is likely due to the statistical power that is provided with next generation sequencing methods. A caveat to consider is that our methods do not allow for the explicit determination of the proportion of pseudouridylated bases that occur at individual sites. Even for highly reproducible sites, it is likely that these sites occur on only a fraction of rRNA molecules and additional studies will be needed for quantitative determination of site frequency. This could potentially be performed with recently described HydraPsiSeq methodology (Marchand *et al*. 2020). Alternatively, nanopore sequencing approaches would potentially allow for determination of Psi sites that occur together on individual rRNA molecules (Begik *et al*. 2021).

In addition to sites on rRNA, we found that the vast majority of mRNAs encoding ribosomal proteins were pseudouridylated. Although the function of the Psi sites on *Drosophila* mRNAs also awaits further analysis, an interesting possibility is that codons containing Psi could favor the incorporation of non-cognate amino acids during mRNA translation (Eyler *et al*. 2019). We found 160 Psi-containing codons across mRNAs encoding for 58 ribosomal proteins. If each of these codons facilitates some degree of non-cognate amino acid incorporation (Eyler *et al*. 2019), this would allow for vast complexity of ribosomal protein isoforms. We hypothesize that such a diversity of ribosomal protein subunits might allow for robustness in ribosomal function across a variety of environmental conditions which could be advantageous for a poikilotherm such as *Drosophila*. Alternatively, ribosomal protein diversity could contribute to cell-type-specific functions of the ribosome, or facilitate translation of particular mRNAs as previously described for other forms of ribosome heterogeneity (Shi *et al*. 2017; Genuth and Barna 2018; Hopes *et al*. 2022).

The most highly pseudouridylated mRNAs (*Yp1*, *Yp2*, and *Yp3*) encode for the Yolk proteins. These transcripts are expressed in fat bodies of the female head which is one of the places where yolk protein biogenesis is thought to occur (Fujii and Amrein 2002). An important aspect of fly vitellogenesis involves synthesis and secretion of the yolk proteins from fat bodies into the hemolymph circulation for transport to the ovaries (Hames and Bownes 1978). High levels of pseudouridylation, especially in the coding region of the Yp transcripts, may facilitate message stability, translation, or possibly plays a role in targeting the mRNA to secretory pathways. Consistent with the latter possibility, the RNA with the highest percentage of Psi was the 7SL RNA which is a component of the Signal Recognition Particle, the key factor in guiding mRNAs to the secretory compartments of the cell. Other mRNAs for secreted and transmembrane proteins that identified in our analysis include rhodopsins (*ninaE*, *rh4*, and *rh6*), *trp*, and odorant-binding proteins (*Obp44a*, *Obp56d*, *Obp56e*, *Obp99c*, and *Obp19D*).

We found that transcripts expressed from the mitochondrial genome (*lrRNA*, *srRNA*, *CoI*, *CoII*, *CoIII*, *AtpAse6*, *AtpAse8*, *ND2*, *ND5*, and *Cyt-b*) and some nuclear-encoded mitochondrial genes (*ATPsynB*, *ATPsynbeta*, *ATPsynC*, *ATPsynF, Cyt-c-p*, *COX6B*, *COX7C*, *UQCR-C2*, *COX5A*, and *porin*) were pseudouridylated. Interestingly, the opal (UGA) cellular stop codon was pseudouridylated in 5 cases on mitochondrial transcripts and we propose that this may facilitate incorporation of tryptophan at ΨGA codon as part of the mitochondrial genetic code. Indeed, our finding that the ΨGA codon is specific to mitochondrially encoded RNAs provides evidence in support of specificity of the Psi sites that we have identified. If our method was to identify spurious sites, we would expect to find ΨGA codons in both nuclear and mitochondrially encoded genes. Other potential Psi sites that were absent from our analysis are further evidence of specificity (Table 1). Notably, we found no instances for Psi on the amber (UAG) or ochre (UAA) stop codons. Indeed, many codons were detected at lower levels than expected if Psi were equally distributed across uridine nucleotides (Table 1).

An interesting pattern is apparent for nuclear and mitochondrial encoded genes and their pseudouridylation of codons for leucine. All of the 7 instances for the UΨA codon (i.e. UUA leucine) were found on mitochondrially encoded mRNAs. We also found evidence for ΨUA modification of the UUA codon, and in 12/13 cases this modification was identified on mitochondrial encoded transcripts. Interestingly, on nuclear-encoded transcripts, the CUG codon for leucine was the codon that was most enriched for Psi. As the CUG codon for leucine is not found at all in mitochondrially encoded genes, all of the instances of CΨG codons are in nuclear-encoded genes (as expected). Thus, in mitochondria there is an enrichment for Psi in the UUA codon for leucine but for nuclear-encoded genes the enrichment is for the CUG codon for leucine. We hypothesize that this use of Psi by the cell may contribute to the appropriate translation of biased codons for leucine on mitochondrial and cellular ribosomes, respectively. We note that leucine (along with serine and arginine) is unusual among amino acids in that it can be encoded by 6 codons (using the canonical bases A, C, U, and G). If one considers that there are 11 possible Psi-containing codons for leucine, the total number of potential codons for leucine expands to 17. The mechanistic importance of this level of diversity for leucine codons poses an interesting puzzle for the future.

Finally, we found that 423 Psi sites were absent from the *RluA-2* mutant RNA and these sites are thus potential targets for the RluA-2 enzyme. The potential RNA targets of RluA-2 included rRNAs, mRNAs, snRNAs, and tRNAs. Among mRNAs, notable targets for RluA-2 included *arrestin2* (*Arr2*) mRNA which was missing 24/42 sites and *neuropeptide like protein 3* (*NPLP3*) was missing 11/14 sites. These 2 targets stand out as potentially important in the previously described hypersensitive nociception phenotypes of *RluA-2* mutant larvae (Song *et al*. 2020). Ribosomal protein mRNA targets of RluA-2 are also of note as 65/160 sites on mRNAs encoding for ribosomal proteins were absent in the mutant background. Interestingly, many of these same ribosomal protein mRNAs showed upregulated expression in the mutant RNA libraries. We look forward to future studies that may further elaborate the impact of RluA-2-dependent pseudouridylation on neuronal and ribosomal function.

## Accession numbers

Psi-sequencing data have been deposited into the Gene Expression Omnibus (GEO) under the accession number GSE213312.

## Supplementary Material

jkac333_Supplementary_Data

## Data Availability

Data generated or analyzed during this study are included in this published article (and its supplementary information files). Psi-sequence data that support the findings of this study have been deposited in the GEO with the accession number GSE213312. Supplemental material available at G3 online.
