# Supplementary material for: Transcriptome-wide analysis of pseudouridylation in Drosophila melanogaster
Source: G3 (Bethesda). 2022 Dec 19;13(3):jkac333. doi: 10.1093/g3journal/jkac333 (PMC9997552; doi:10.1093/g3journal/jkac333)
Supplement: jkac333_Supplementary_Data [file jkac333_supplementary_data.zip › Supplemental_Material_Legends_G3-2022-403856.docx]

**Supplemental Materials**

**Supplemental Figures**

**Figure S1.** Optimization of CMCT treatment conditions for Psi-seq library preparations. The polyA-selected RNA was subjected to either fragmentation (“Fragmented before treatment”) or not (“Non-fragmented before treatment”) prior to the CMC/mock derivatization. The derivatization was carried out with CMC/mock treatment for 1hr or 18hr at 37°C (“1hr CMC/mock” or “18hr CMC/mock”), followed by alkaline treatment for 6hr at 37°C or 75°C (“37°C hydrolysis” or “75°C hydrolysis”). The Y axis represents the termination fold change while the X axis is the nucleotide position on the RNA spike-in sequence. The termination fold change from the only pseudouridine at position 43 on the RNA spike-in is marked with a red line whereas those from other sites are presented as black lines. The optimum condition producing the highest termination fold change for Psi site and lowest background is framed with a dashed red rectangle.

**Figure S2.** The termination fold change (y axis) distribution over the large subunit (LSU) of cytoplasmic rRNA in *Drosophila melanogaster* (x axis). The Psi sites identified are called with the threshold of appearing in 6 or all 7 *w^1118^* libraries (high reproducible site, red), 4 or 5 libraries (intermediate reproducible site, blue), and 2 or 3 libraries (low reproducible site, green) from both sets of the experiments. The previously reported Psi sites on LSU identified by Ofengand et al (1997) were indicated by the gray vertical lines.

**Figure S3.** Consensus sequence for the RluA-2 target sites. The X axis is the +/- 10 nucleotide sequence flanking the T site where the RNA bears the pseudouridylation modification (0 position). The Y axis represent the bits score. The overall probability of a nucleotide is represented by the height of the letter.

**Figure S4.** Euclidean distances of *w^1118^* and *RluA-2* mutant mock libraries clearly demonstrate the clustering of samples based on the genotypes. Color key indicates level of similarity between libraries.

**Supplemental File S1**

**Table S1**. A summary of the Psi-seq libraries (Table S1) and an overview of contents included in supplemental tables (Table S2 - Table S8) for transcriptome-wide mapping of pseudouridylation and transcriptome expression analyses in *Drosophila melanogaster*.

**Table S2**. The quality features of the sequencing data used for Psi-seq and transcriptome expression analyses in *Drosophila melanogaster*.

**Table S3**. Psi sites identified using Psi-seq on cytoplasmic and mitochondrial rRNA in *Drosophila melanogaster*. Note the cytoplasmic LSU rRNA sites reported previously are labeled as “Ofengand site” in column H.

**Table S4**. Psi sites detected on other types of ncRNA with Psi-seq, including tRNA, snRNA, snoRNA, and other ncRNA in *Drosophila melanogaster*.

**Table S5**. Psi sites detected on protein-encoding mRNAs with Psi-seq in *Drosophila* *melanogaster*.

**Table S6**. Potential RluA-2 pseudouridylation targets in the adult head of *Drosophila melanogaster* with Psi-seq. Pseudouridylation sites from *RluA-2* (n=4) and *w^1118^* (n=4) samples from the same set of experiment (Exp 2) were compared and those sites which are called in at least two libraries from both of the experiments (Exp 1 and Exp 2) in *w^1118^* but absent from *RluA-2* were listed as the putative RluA-2 targets*.*

**Table S7**. Differentially expressed genes in *RluA-2* compared to the genetic background iso*w^1118^* in the adult head of *Drosophila melanogaster.* The mock samples from each Psi-seq data set were used for differential expression analysis between the 4 replicates of *RluA-2* samples and 4 replicates of *w^1118^* samples. Differentially expressed genes were detected using DeSeq2 with the threshold of adjusted *p* value with IHW correction <0.05*.*

**Table S8**: A list of differentially expressed genes between *RluA-2* and *w^1118^* which overlap with genes containing RluA-2 target Psi sites.
