# Supplementary figures and images for: Transcriptome-wide analysis of pseudouridylation in Drosophila melanogaster
Source: G3 (Bethesda). 2022 Dec 19;13(3):jkac333. doi: 10.1093/g3journal/jkac333 (PMC9997552; doi:10.1093/g3journal/jkac333)

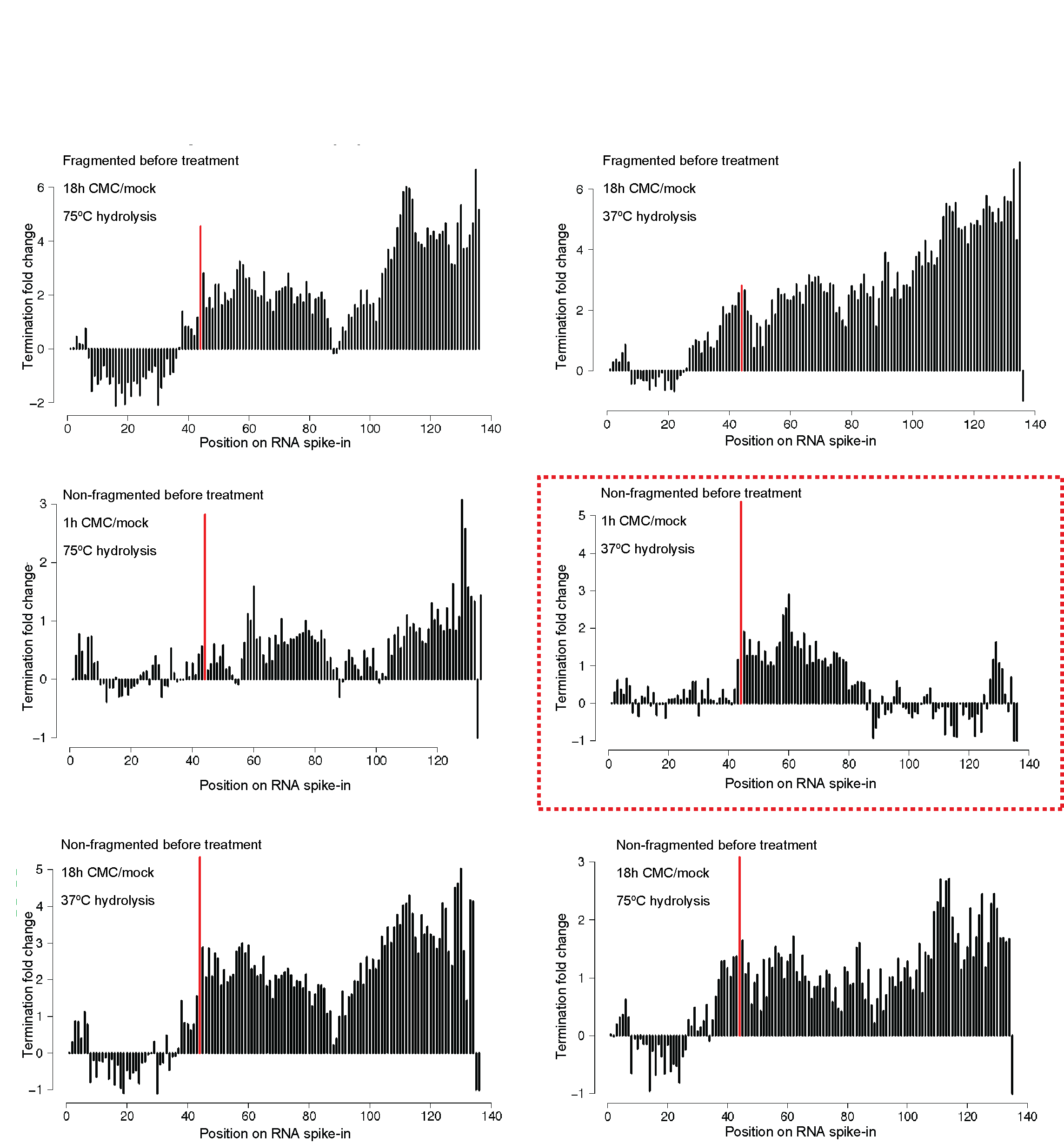

Supplement: jkac333_Supplementary_Data [file jkac333_supplementary_data.zip › Figure_S1_G3-2022-403856.tif]

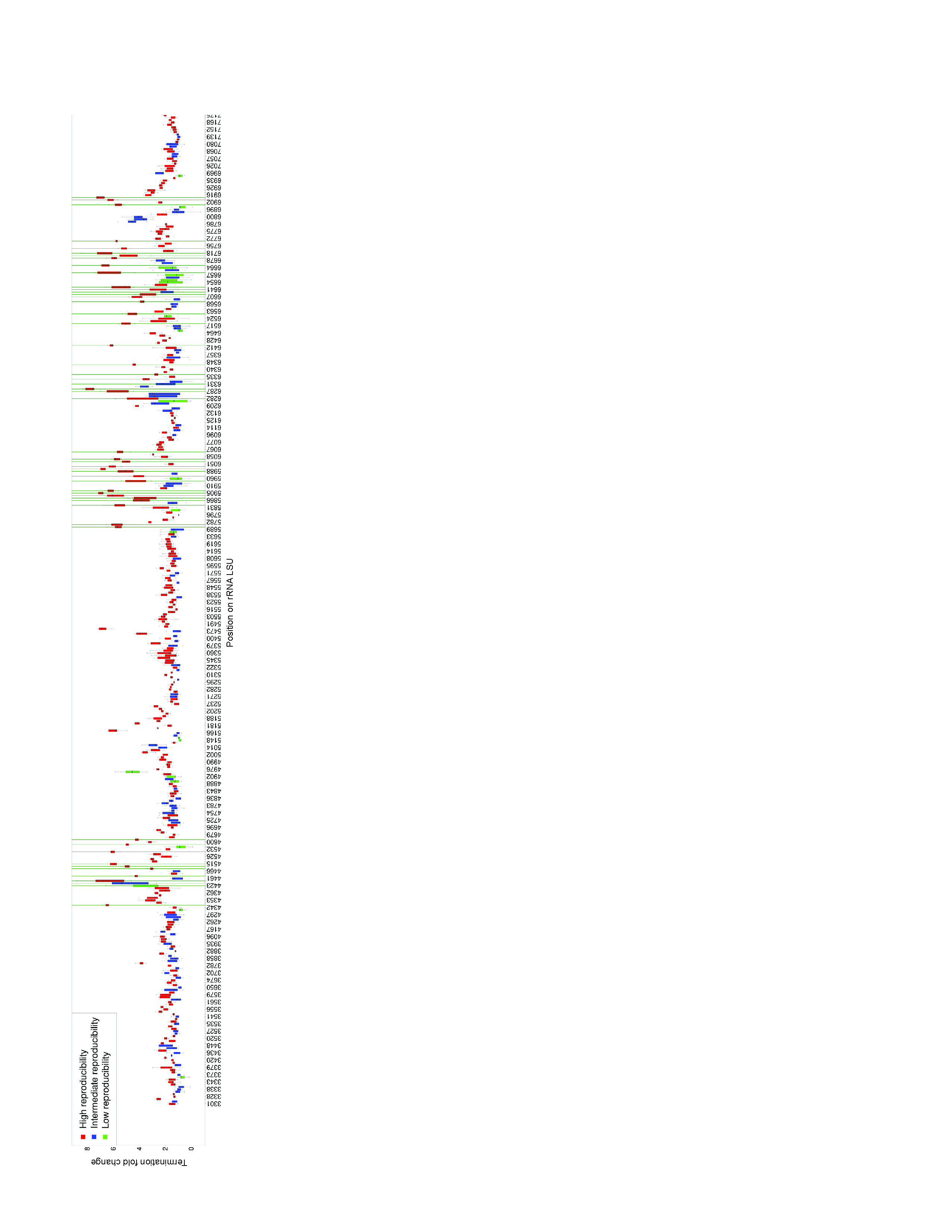

Supplement: jkac333_Supplementary_Data [file jkac333_supplementary_data.zip › Figure_S2_G3-2022-403856.tif]

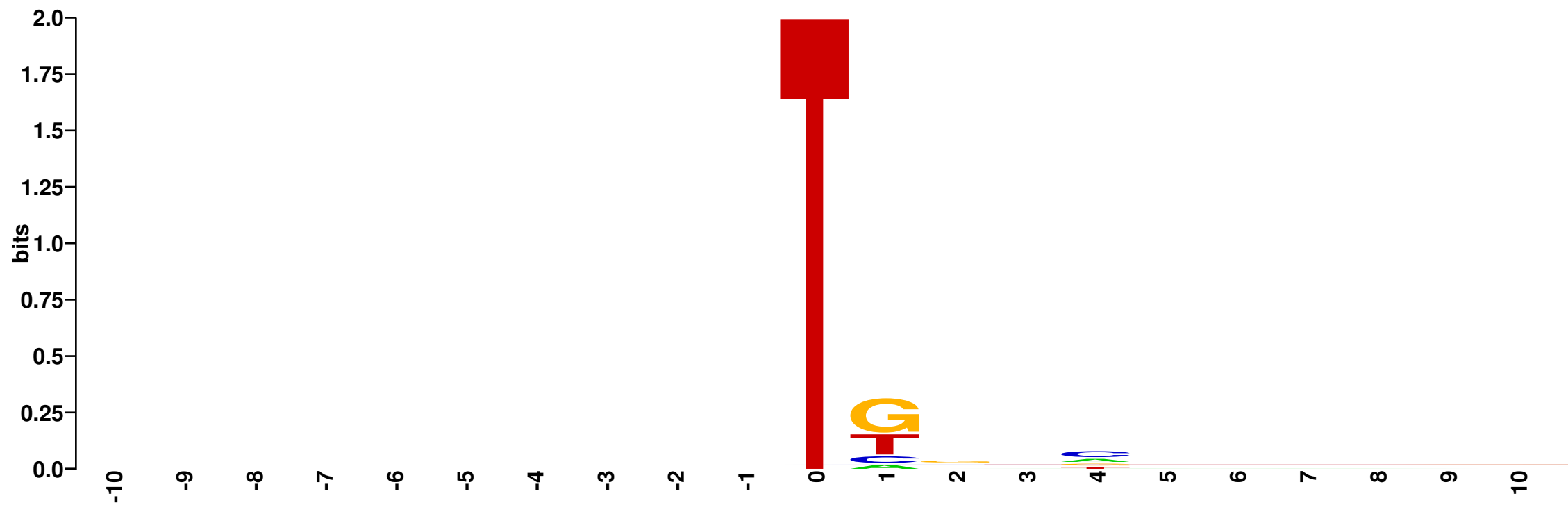

Supplement: jkac333_Supplementary_Data [file jkac333_supplementary_data.zip › Figure_S3_G3-2022-403856.pdf]

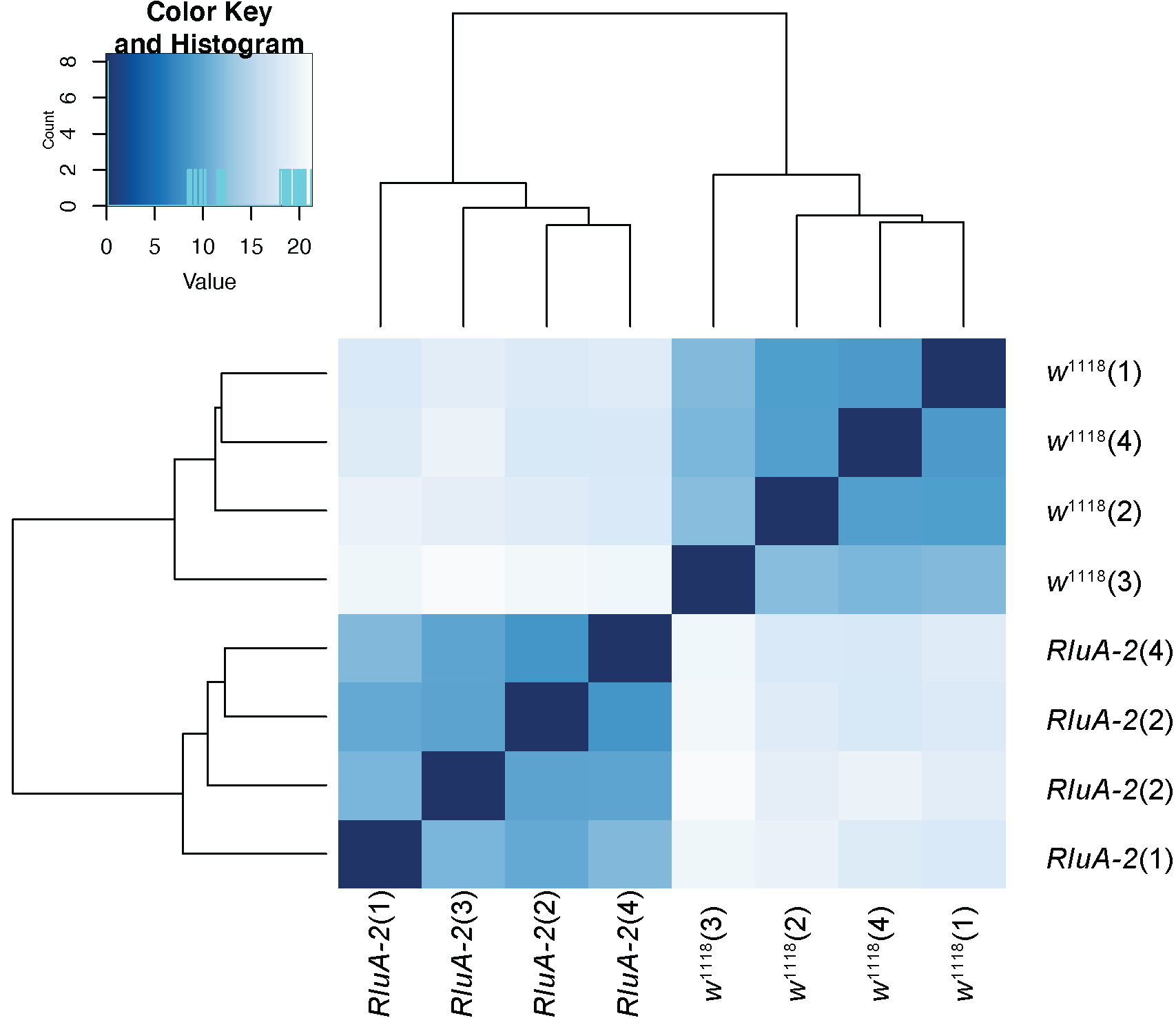

Supplement: jkac333_Supplementary_Data [file jkac333_supplementary_data.zip › Figure_S4_G3-2022-403856.tif]
